# Near-infrared reflectance imaging of neovascularization in proliferative diabetic retinopathy

**DOI:** 10.1186/s40942-020-00263-8

**Published:** 2020-11-23

**Authors:** Sara Vaz-Pereira, Manuel Monteiro-Grillo, Michael Engelbert

**Affiliations:** 1 Department of Ophthalmology, Centro Hospitalar Universitário de Lisboa Norte, EPE - Hospital de Santa Maria, Avenida Professor Egas Moniz, 1649-035 Lisbon, Portugal; 2 Department of Ophthalmology, Faculdade de Medicina, Universidade de Lisboa, Lisbon, Portugal; 3 ALM – Oftalmolaser, Lisbon, Portugal; 4 Vitreous Retina Macula Consultants of New York, New York, NY USA; 5 LuEsther T. Mertz Retinal Research Center, Manhattan Eye, Ear and Throat Hospital, New York, NY USA; 6 Department of Ophthalmology, New York University School of Medicine, New York, NY USA

**Keywords:** Diabetes mellitus, Diabetic retinopathy, Diagnostic imaging, Near-infrared reflectance imaging, Optical coherence tomography, Proliferative diabetic retinopathy, Retinal neovascularization

## Abstract

**Background:**

Blood is one of the main absorbers in the near-infrared spectrum and thus retinal vessels appear dark in near-infrared reflectance (NIR) images. Proliferative diabetic retinopathy (PDR) is characterized by abnormal neovascularization which also absorbs light and appears dark against a lighter fundus background. We analyzed neovascularization in PDR using NIR imaging, by observing changes in the neovascular complexes (NVCs) contrast and reflectivity over time.

**Methods:**

Retrospective case series of 20 eyes of 17 patients with PDR who underwent NIR imaging with optical coherence tomography (OCT) using the Spectralis System. NVCs presence and activity was determined using clinical, tomographic and angiographic criteria. At baseline, all NVCs were qualitatively graded in the NIR image into 3 groups (absent, present and inactive and present and active) and their evolution over time was registered as progression, regression or same status.

**Results:**

Twenty-seven NVCs were imaged, of which, 52% were neovascularization of the disc (NVD) and 48% were elsewhere (NVE). Consecutive NIR images were obtained from baseline to up to 5 time-points with a mean follow-up of 3.2 ± 1.7 years. All eyes underwent laser treatment and 30% had additional intravitreal therapy. Using NIR imaging, NVCs were classified at baseline as absent, present and inactive and present and active, respectively in 11, 4 and 85% of cases. NIR identified active neovascularization as hyporeflective irregular dark vessels originating from the retinal venules in NVE or from the disc in NVD. In all groups during follow-up, progression was identified as the development of new vascular hyporeflective dark fronds while regression was shown by reduced dark perfusion. Five eyes developed a wolf’s jaw configuration with vascular hyporeflective new vessels and hyperreflective tissue from extensive fibrosis. Fibrosis was more apparent in later images, reaching 86%. In 3 cases (11%), the NVC was no longer seen in NIR, although was still identifiable on OCT over the NVC area.

**Conclusions:**

NIR is a non-invasive imaging modality commonly performed alongside OCT and frequently overlooked which can be useful to evaluate NVCs in PDR. Changes in NVC contrast and reflectivity due to blood perfusion can help in the detection and monitoring of diabetic proliferative disease and aid clinicians in daily practice.

## Background

Diabetic retinopathy (DR) is a major cause of blindness with the total number of individuals affected by diabetes mellitus (DM) rising worldwide [1]. Vision loss can occur either by significant diabetic macular edema or complications of proliferative diabetic retinopathy (PDR) [2]. The latter is estimated to affect around 7% of people with diabetes, the prevalence increasing with disease duration and in type 1 diabetes [2]. The presence of neovascularization is the hallmark of PDR and it can be located on the optic disc (NVD) or elsewhere in the retina (NVE) [3]. In advanced diabetes the abnormal neovascular complexes (NVCs) can bleed and contract contributing to hemorrhagic and/or tractional complications [3, 4].

Early and prompt recognition of the presence and progression of PDR is, therefore, essential and clinicians can nowadays use a multimodal approach [5]. Established technologies to determine the presence of neovascularization are color fundus photography [6, 7], fluorescein angiography (FA) [8, 9], optical coherence tomography (OCT) [10–15] and, more recently, OCT angiography (OCTA) [15–21]. Fundus photography is mostly used for documentation of clinical examination and for clinical trials purposes while FA is important to identify areas of vascular leakage and non-perfusion [5]. Using structural OCT, NVEs are identified as tissue of medium to high reflectivity that breaches the internal limiting membrane (ILM) and NVDs as tissue sitting above or protruding from the disc [13–15]. OCTA has the advantage of incorporating the en face image, which shows the morphology of the NVCs, to the corresponding OCT-B scan with flow overlay which helps to evaluate disease activity [15, 16].

In daily clinical practice with busy clinics, performing all these assessments can be rather time-consuming including moving the patient to several different machines. Ideally, maximum information is obtained with the least necessary testing. This has become all the more important with the COVID-19 pandemic challenging how we adjudicate time and resources to patients in the interest of social distancing and minimizing time spent in physical spaces with limited air circulation. Currently, most commercial OCT devices incorporate a high-contrast near-infrared reflectance (NIR) image acquired in combination with the structural OCT. NIR is a non-invasive imaging modality that has proven useful to study subretinal structures [22], with its main application in neovascular age-related macular degeneration [23–25]. It has also been of interest in other retinal diseases, although not commonly used as a regular clinical test [26]. Specifically in DR, it has been used to quantify and locate cysts in cystoid macular edema [5, 27] and we have reported a case where NIR was useful in the evaluation and follow-up of PDR [28]. It is our impression that information of the NIR image is frequently disregarded, but that this information is useful in busy clinics, and may even obviate the need of extra testing such as fundus photography or OCTA. To interpret the images in the context of PDR it is important to consider that blood is one of the main absorbers in the near-infrared spectrum and therefore retinal vessels appear dark in NIR images, while collagen and fibrotic tissue appear bright [25, 28]. PDR is characterized by the growth of abnormal vessels which also absorb light and appear dark against a lighter fundus background [24, 28].

We present a novel approach on the evaluation and follow-up of PDR using NIR imaging. Our observations show that NIR is useful in routine clinical examination and can help in the detection and monitoring of diabetic proliferative disease aiding clinicians in clinical practice.

## Methods

This was a retrospective case series including eyes from patients with PDR from the medical retina clinic from one referral center – Hospital de Santa Maria, Lisbon, Portugal. Patients with PDR in at least one eye were identified using a database search and then shortlisted to only include the ones who had NIR imaging of NVCs obtained at the time of OCT acquisition using the Spectralis scanning laser ophthalmoscope (Heidelberg Engineering, Heidelberg, Germany) as part of routine clinical examination. NVCs presence and activity was determined using clinical, tomographic and angiographic criteria. Exclusion criteria included other causes of proliferative retinopathy, previous vitrectomy and significant media opacities.

High-contrast digital NIR images with a field of view of 30° were acquired using the 830 nm diode laser of the Spectralis System. The images were captured at the time of acquisition of the macular or NVC OCT scans and then retrospectively reviewed and analyzed. A quality check was initially performed to confirm adequate focus and exclude images with eye motion artifacts.

Retinal neovascularization was classified as “NVE” and disc neovascularization or within 1 disc diameter from its margin as NVD [7]. Using structural OCT, NVDs were classified morphologically as sitting or protruding from the disc and the NVE as “flat”, “ forward” and “tabletop”, as previously described [14, 15]. All NVCs were qualitatively graded in the NIR image at baseline as (1) absent, if not apparent, meaning no changes in reflectivity were noted, (2) present but inactive if the new vessel structure was apparent and of medium to high reflectivity, respectively same reflectivity of the normal fundus or brighter than the fundus, as seen in atrophic regions or in collagen/fibrotic tissue, but without perfusion, defined as the presence of a hyporeflective blood column that appeared dark as the normal retinal vessels and (3) present and active if apparent and with an identifiable hyporeflective blood column [25, 28]. The changes at follow-up visits were documented as same status (no change), progression (additional hyporeflective vascular fronds identified) or regression (fewer hyporeflective vascular fronds identified). The presence of associated fibrosis defined as hyperreflective tissue or new vitreous hemorrhage (hyporeflective) was also noted [25, 28].

Statistical analysis was performed using Microsoft Office Excel 2019 for Mac version 16.24 and SPSS version 24.0 (IBM Corp, Armonk, NY). Data were analyzed with frequency and descriptive statistics. Independence chi-square and Fisher’s exact tests were performed for the categorical variables. *P* value was considered statistically significant when < 0.05.

This study was approved by the center local ethics committee and was conducted in accordance with the ethical principles of the Declaration of Helsinki and International Conference on Harmonization Good Clinical Practice Guidelines. Informed consent was obtained from all subjects.

## Results

Twenty eyes of 17 diabetic patients were evaluated. Ten (59%) patients were male, with a mean age ± standard deviation of 56 ± 14 years, and 14 (82%) were white while 3 (18%) had African origin. Five (29%) had type 1 DM and 12 (71%) had type 2 DM.

Twenty-seven NVCs were imaged, of which 14 (52%) were NVD and 13 (48%) were NVE. Six eyes had both NVD and NVE imaged and in one eye 2 NVEs were documented. Consecutive images were obtained from baseline to up to 5 time-points, with a mean follow-up of 3.2 ± 1.7 years. Fourteen (70%) eyes underwent laser treatment and 6 (30%) eyes a combination of laser and intravitreal anti-VEGF treatment.

### Tomographic analysis of NVCs

Of the 14 NVDs, 3 (21%) were classified as sitting over the disc and 11 (79%) as protruding from the disc. Regarding NVEs, 2 (15%) were flat, 6 (46%) were forward and 5 (38%) were tabletop.

### NIR image analysis of NVCs

At baseline, 3 (11%) of the 27 NVCs were classified as absent, 1 (4%) as present but inactive and 23 (85%) as present and active. Hyperreflective fibrotic tissue was identified at baseline in 7 (26%) of NVCs, respectively in 1 (100%) of the NVCs considered present and inactive and in 6 (26%) of the classified as present and active. No association was found between activity and level of fibrosis at baseline (p = 0.29).

### Group of absent NVCs at baseline

**Fig. 1:**
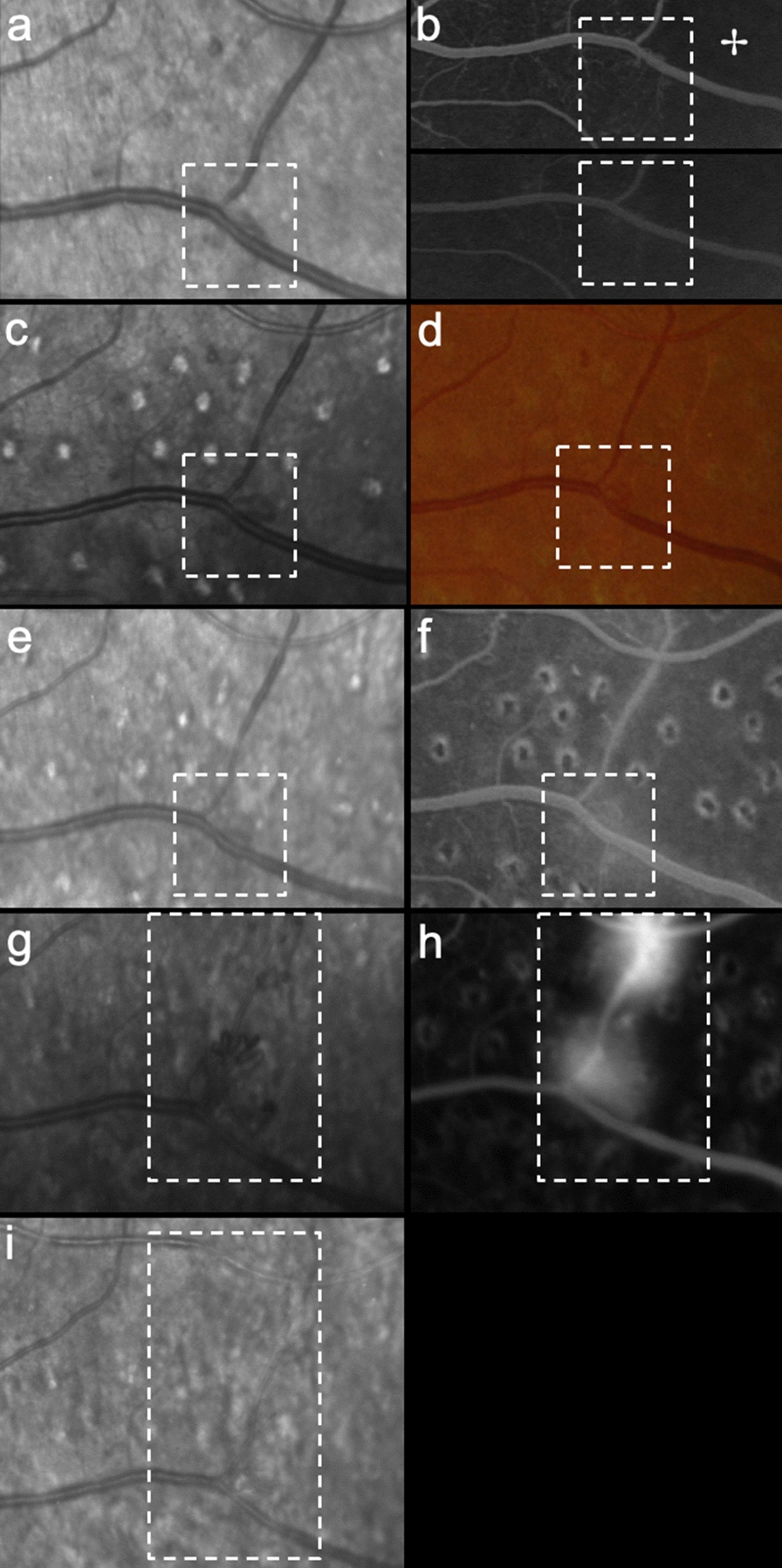
NIR sequence of IRMA growing into NVE over 4 years. Baseline NIR **a** shows hyporeflective irregular vessels arising from the inferior temporal vein (box). Corresponding fluorescein angiography **b** in early (top) and late (bottom) frames is in accordance with IRMA as no late leakage. Note the associated areas of non-perfusion (✢). The patient was started on panretinal photocoagulation as there were other areas of proliferation. At 6 months follow-up, a change in morphology with enlargement of the complex (box) in the NIR image can be observed (**c**). Matching color fundus photograph **d** and clinical examination were compatible with NVE. Laser treatment was continued and on a subsequent visit, the vascular complex was less apparent, suggesting regression **e** and the angiogram showed only mild late leakage (**f**, box). The NVE remained stable until 2 years from (**f**), when significant progression was identified in the NIR image **g** with new hyporeflective fronds (box), in accordance with NVE reactivation and growth. Complementary fluorescein angiography **h** shows late leakage with 2 NVE areas, clearly more active than in **(f**). With additional laser treatment, NIR **i** 4 months from **g** shows regression and thinning of the hyporeflective structures reflecting NVE regression, which was corroborated by clinical examination

**Fig. 2:**
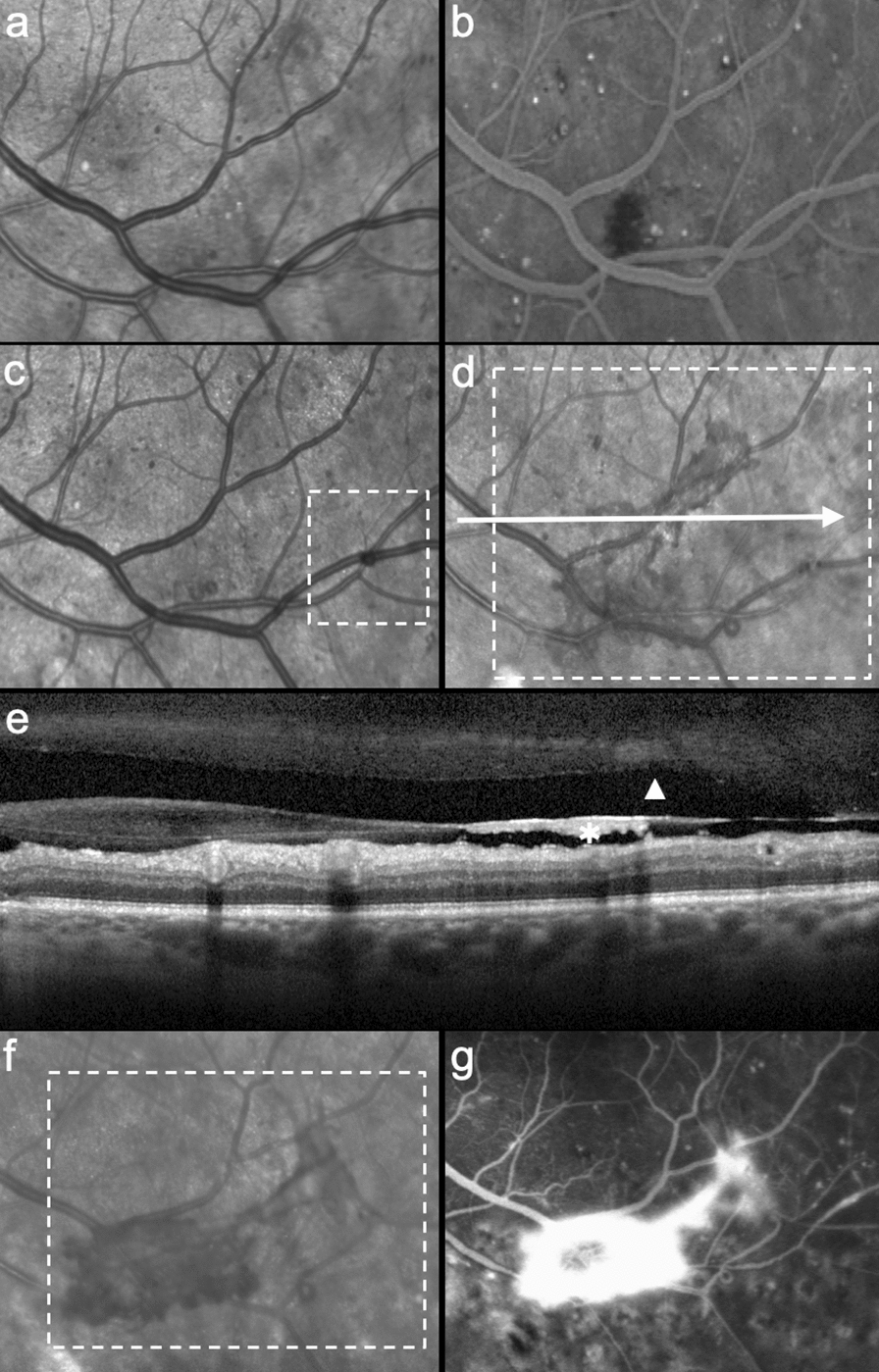
NIR series of newly developing NVE. Baseline NIR **a** and fluorescein angiography **b** respectively showing no reflective changes and no leakage. At 6 months follow-up, observe a new hyporeflective change arising from a secondary inferior branch venule (**c**, box). The patient was then lost to follow-up and at the returning visit 4 years from baseline, 2 vascular loops and a fully developed neovascular frond (box) were documented in NIR **d** as hyporeflective irregular vessels. Optical coherence tomography **e** at the level of the arrow in **d** shows the neovascular tissue (✱) growing along the posterior hyaloid outer surface without invading the premacular bursa (**▲**). Despite panretinal photocoagulation, **f** shows progression of the NVE with more hyporeflective fronds and fluorescein leakage (**g**)

Three (11%) NVEs of the 27 NVCs were classified as absent at baseline, meaning they were not apparent in NIR image, which was corroborated by clinical, tomographic and/or angiographic criteria (Figs. 1 and 2). The mean ± standard deviation (SD) follow-up of this subgroup was 5.1 ± 0.9 years and all NVCs had at least 3 time-point evaluations from baseline (range 3–4).

**Fig. 3:**
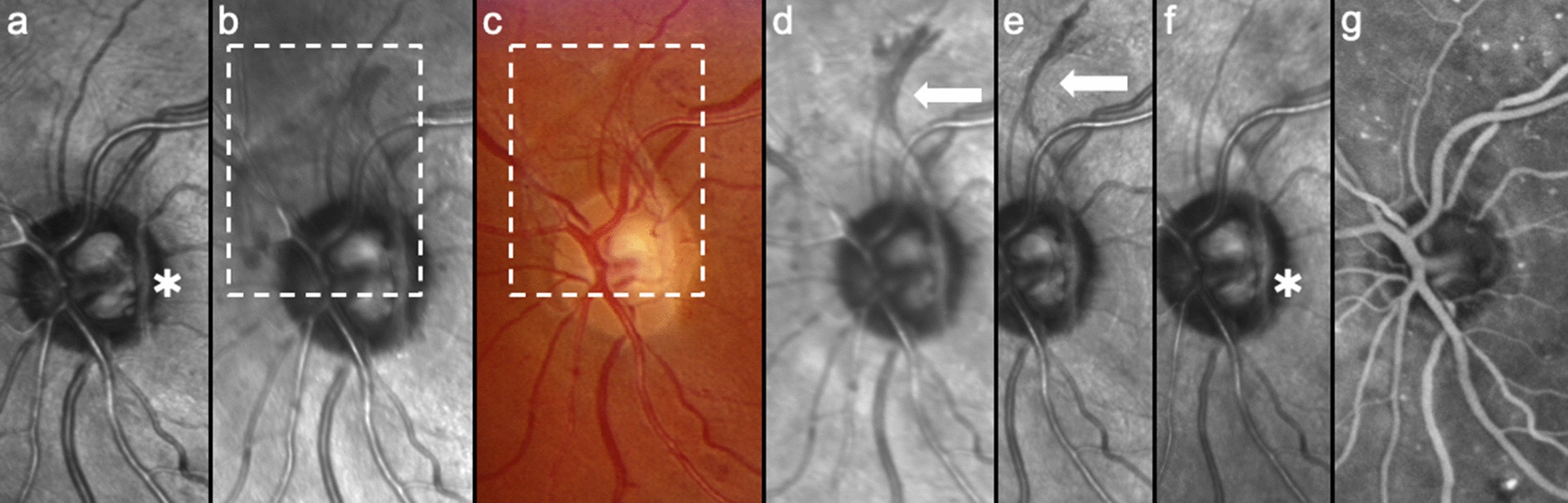
Sequence of NVD over 4 years showing reactivation and subsequent regression. Baseline NIR **a** shows an inactive NVD with hyperreflective fibrotic component (✱). Note reactivation in the NIR image **b** with the development of new vascular hyporeflective dark fronds (box) and matching color fundus photograph **c** where the vascular component is easily identified (box). After panretinal photocoagulation, there is some involution of the nasal neovascular tissue, albeit with progression at the level of the arrow (**d**). With continuation of laser treatment, there is regression of the neovascular complex (**e**–**f**), shown by reduced dark perfusion. In **f** there is only hyperreflective avascular fibrotic tissue (✱) and no leakage on fluorescein angiography (**g**)

At the first follow-up, the neovascular complex became apparent in all cases (100%), by the identification of new hyporeflective irregular fronds on NIR image, and was graded as progression. One developed from a preexisting IRMA (Fig. 1) and 2 originated from a secondary temporal branch venule. In one of these cases (Fig. 2), associated venous loops were documented. At the 2^nd^ follow-up visit, 2 (67%) of the complexes were classified as having progressed while 1 (33%) regressed. At the last visit, 2 of NVEs had regressed, while one kept progressing despite panretinal photocoagulation (Fig. 3). During follow-up, the development of hyperreflective fibrotic tissue on NIR was documented in this case (Fig. 3) with the development of a wolf’s jaw fibrovascular configuration. No hemorrhaging was identified in these eyes. Regression was observed as involution of the hyporeflective vascular fronds previously identified (Fig. 1).

### Group of NVCs present but inactive at baseline

One (4%) of the NVCs was categorized as present but inactive at baseline (Fig. 3). The neovascular structure was identifiable by changes in reflectivity – medium high due to the presence of the fibrous skeleton of the vessel, but no hyporeflective filling from perfusion was observed. The follow-up of this NVC was 3.7 years and had 5 evaluations from baseline. At the 1st three time-visits, progression with development of new vascular hyporeflective dark fronds was identified with subsequent regression after panretinal photocoagulation treatment. At the last visit, only the hyperreflective avascular fibrotic tissue was observed and there was no fluorescein leakage, in accordance with disease inactivity (Fig. 3).

### Group of NVCs present and active at baseline

**Fig. 4:**
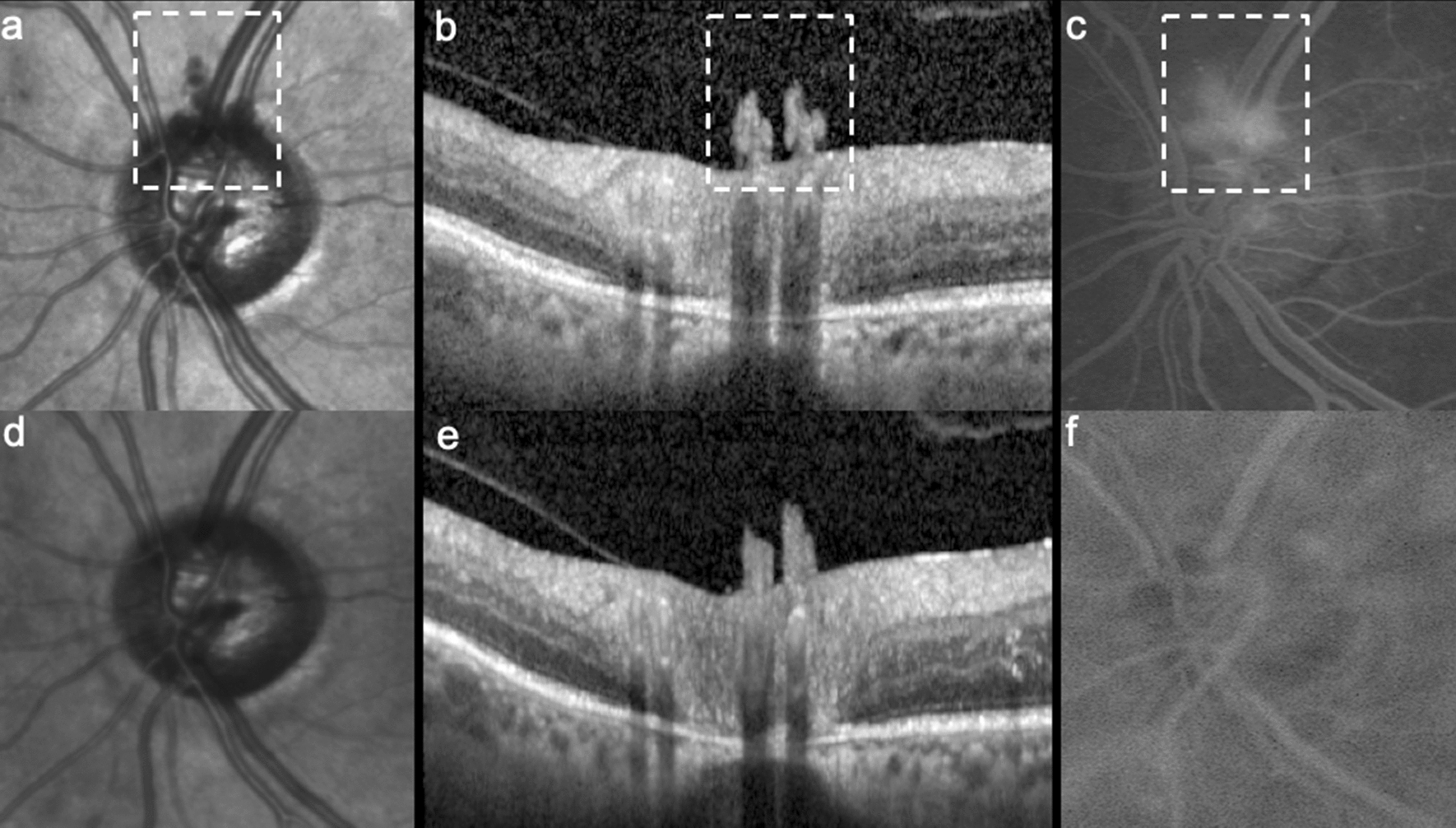
NIR sequence of active NVD with regression after treatment. Note the presence of hyporeflective dark vessels originating from the disc in NIR **a**, which protrude from the disc in the structural OCT (**b**, box) and leak on fluorescein angiography (**c**, box). After laser treatment, the vessels disappeared in both NIR (**d**) and fluorescein angiography (**f**), although their structure is still apparent on OCT (**e**)

**Fig. 5:**
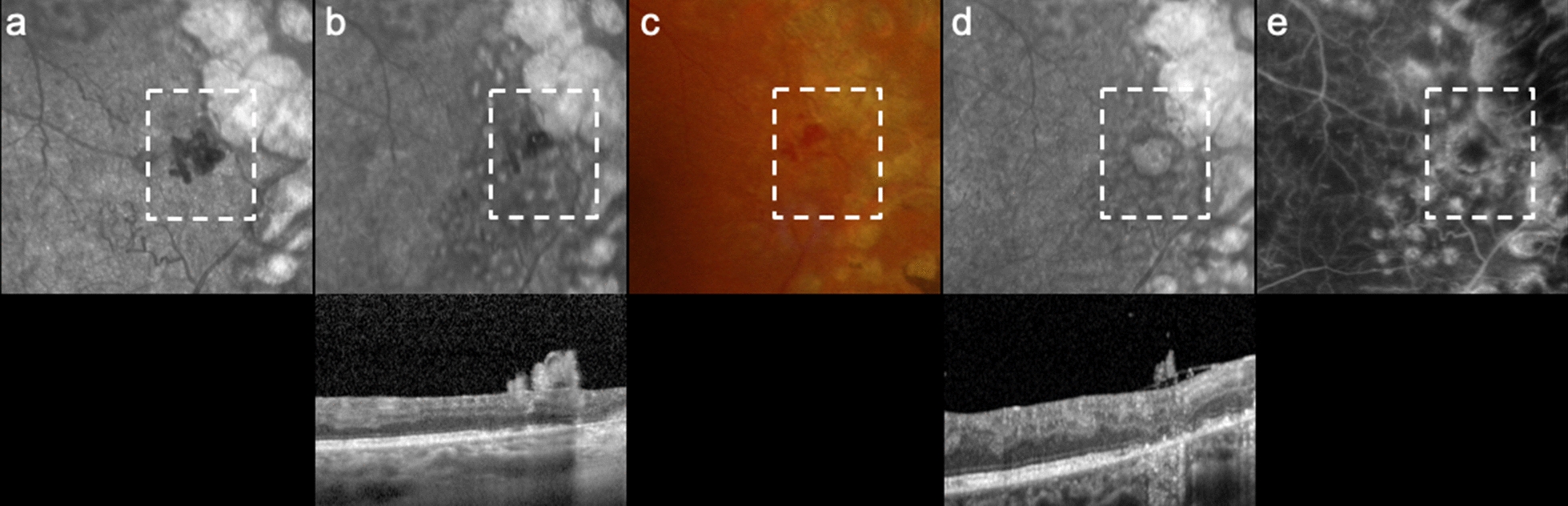
NIR series of regressing NVE. Baseline NIR **a** shows the presence of hyporeflective irregular dark vessels originating from the retinal venules in the temporal macula (box). With focal laser treatment, there is regression shown by reduced dark perfusion in the NIR (**b**, top; box). OCT through the lesion **b**, bottom, shows ILM breaching characteristic of NVE. Note the perfused vessels in contemporaneous color fundus photograph (**c**, box). With continuation of treatment, the NVE disappears in NIR **d**, top; box, although its structure, albeit smaller, is still identifiable on OCT (**d**, bottom). Corresponding fluorescein angiography **e** shows no leakage, confirming involution of the NVE (box)

**Fig. 6:**
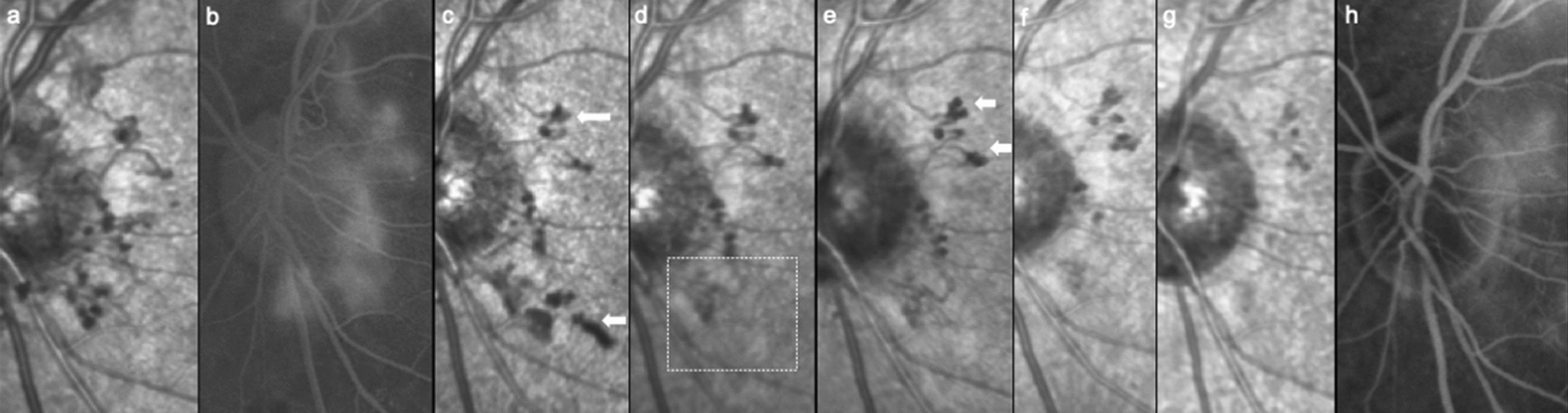
NIR sequence of NVD imaging over 4 years. Baseline active NVD on both NIR **a** and fluorescein angiography **b**. NIR displays hyporeflective neovascularization in greater detail compared to early phase angiography. At 6 months from baseline, it is possible to identify in NIR **c** new hyporeflective vascular buds (arrows), corresponding to neovascular growth that involuted after laser treatment (**d**), shown by the reduced dark perfusion of the vessels in NIR (box). During follow-up, NIR **e** shows subsequent progression (arrows) and regression (**f**, **g**), after laser. Fluorescein angiography **h** at the same time as **g** is in accordance with NVD regression

**Fig. 7:**
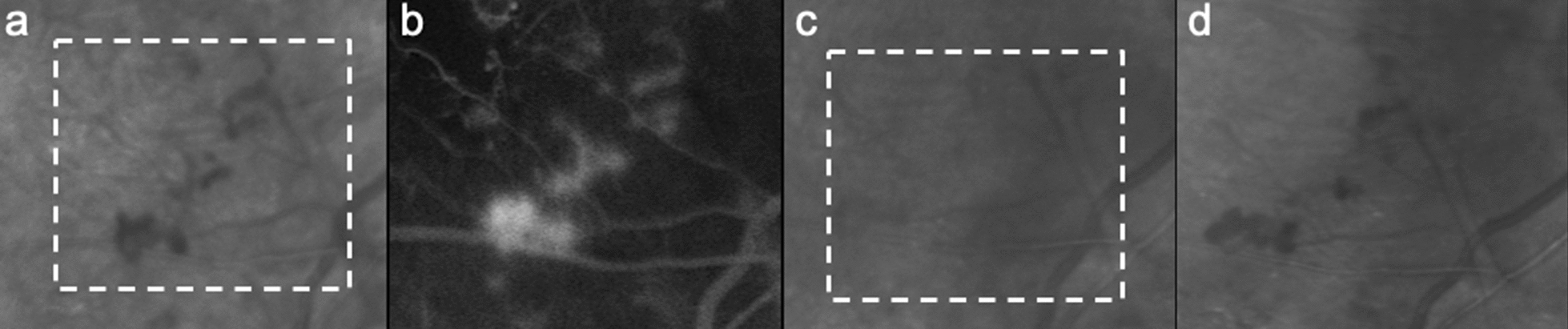
Baseline infero-macular NVEs (box) on NIR **a** and fluorescein angiography (**b**). Note complete regression **c** after intravitreal anti-VEGF treatment (box) and subsequent recurrence, with the presence of new hyporeflective buds, 2 months after treatment (**d**)

This cluster was the largest in our series and included 23 (85%) of the NVCs from 17 eyes. Active NVCs were identified in NIR as irregular dark vessels with an identifiable hyporeflective blood column originating from the retinal venules in NVE or from the disc in NVD (Figs. 4, 5, 6 and 7). The NVCs in this subgroup (57% NVDs, 43% NVEs) had a mean ± SD follow-up of 2.9 ± 1.6 years and all had at least 2 time-point evaluations from baseline (range 2–5). Eleven (65%) eyes underwent panretinal photocoagulation and 6 (35%) a combination of laser and anti-VEGF therapy.

NIR regression shown by reduction of the hyporeflective blood column, i.e. dark perfusion (Figs. 4 and 5), was observed in the 1st follow-up visit in 8 (35%) of cases, in 12 (52%) of cases in the 2nd follow-up and in 8 (73%) of cases at the 4th visit in agreement with treatment sessions. Progression with new vascular dark hyporeflective fronds (Fig. 6) was documented in respectively 8 (35%) cases at first visit, 8 (35%) at the second visit, and 1 (9%) at the 4th visit observation. Observe the presence of vascular active buds (Fig. 6) and their progression and regression after treatment, easily noted by the changes in contrast and reflectivity due to perfusion of the NVC. The mean of regression and progression for all evaluations was, respectively, 49% and 36%. Figure 7 illustrates regression of the NVC hyporeflective vessels after anti-VEGF treatment with recurrence documented by the reappearance of the hyporeflectivity of the NVC after anti-VEGF wash-out. A mean of 16% of NVCs remained stable for the period of evaluation.

The development of hyperreflective fibrosis was noted in 5 (22%) of NVCs and new vitreous hemorrhage was observed in 6 (26%) eyes.

### Additional findings

Five (25%) eyes developed a wolf’s jaw fibrovascular configuration with vascular hyporeflective new vessels and hyperreflective tissue from extensive fibrosis. From these eyes, 4 (80%) were submitted to pars plana vitrectomy along with one eye with persistent vitreous hemorrhage (total of 5 eyes–25%). The presence of a wolf’s jaw was more frequent in type 1 DM (p = 0.005), with florid DR. Fibrosis was more apparent in later images, with a mean of 31% in the first two visits and a mean of 86% at the last 2 visits.

Additionally, although in 3 cases (11%) the NVC was no longer apparent on NIR, the fibrotic skeleton was still identifiable on structural OCT over the NVC area (Figs. 4 and 5).

## Discussion

Near-infrared reflectance imaging, which uses infrared light to illuminate the retina, has been shown useful in imaging through small pupils, media opacities, blood, pigment, exudation and subretinal fluid [5]. Therefore, most studies have shown its use in age-related macular degeneration [23–25], with just few reports on DR and PDR [14, 27, 28].

We have previously published a medical image on this topic [28] and in this larger study we analyzed neovascularization in PDR using NIR imaging, by observing changes in contrast and reflectivity of the neovascular blood column during different time-points. Thirty-seven NVCs from 20 eyes were retrospectively evaluated and divided in 3 groups at baseline (absent, present and inactive and present and active) according to the identification of the neovascularization.

The main group was the one where the NVCs were found present and active in the NIR image. We found that the NVC hyporeflective blood column could be clearly observed and that its progression or regression, by the identification of additional or fewer hyporeflective vascular fronds, respectively, could be determined by an attentive observation of the NIR [28]. The regression was related with disease treatment, such as panretinal photocoagulation and anti-VEGF treatment. In both cases, reduction in the hyporeflective branches or thinning of blow column could be identified, in some cases with subsequent reactivation as after anti-VEGF wash-out. Laser and anti-VEGF are known to modulate neovascularization in PDR and these changes, which can be seen in FA by reduced leakage and in OCTA by the reduction of the vessels on the en face image and flow signal on the co-registered B-scan, can also be appreciated using NIR [15, 21, 29–31].

Regarding the NVCs categorized as absent at baseline, we observed newly developed hyporeflective irregular fronds on NIR, that were definitely not present at baseline images and complementary imaging. It was possible to document that the NVEs developed from preexisting IRMA and from secondary venules with associated venous loops in one case. IRMA have long been proposed as precursors of NVE [15, 32, 33] and eyes with venous loops have been considered to have an extremely high probability of being in some stage of proliferative retinopathy [34]. The identification of these vascular abnormalities was possible with the high-contrast digital NIR alongside structural OCT, complementing clinical examination.

The wolf’s jaw configuration of a perimacular ring of fibrovascular preretinal tissue [14, 35] was more frequent in type 1 DM with florid DR, which needed surgical intervention [2, 36]. NIR imaging showed the neovascularization as hyporeflective fronds and the fibrosis as hyperreflective tissue [25, 28]. The hyperreflective fibrotic component increased after laser treatment, as it likely relates with the scaring and retraction that accompanies the involution of the neovascularization [13]. Additionally, even with regression of the vascular component, with the NVC becoming unapparent on NIR, the fibrotic skeleton of the vessels was still identifiable on structural OCT. The fibrovascular proliferative tissue forms when abnormal new vessels grow in response to ischemia. In the early PDR stages, the abnormal vessels are devoid of fibrotic tissue, which then develops in more advanced stages and fills the intervascular spaces, ultimately resulting in a dense white scar. Also, anti-VEGF and laser therapies alone can contribute to retinal scarring and fibrosis [37]. It is, therefore, not surprising that the fibrotic tissue remains when there is regression of the mature neovascularization [37, 38].

Limitations of this study include its retrospective nature, the sample size and selection bias as most NVCs were captured within the central 30º. On the other hand, we believe to have shown the potential of NIR, which is captured at the same time of OCT in most available commercial devices, obviating the need for additional testing in busy clinics. Although a wide range of techniques are currently useful to evaluate PDR, such as OCT/OCTA, the invasive FA and their widefield modalities [15], and we agree that multimodal imaging is important in the management of retinal diseases, often not all imaging techniques are available in clinics and even when they are, acquiring all the multimodal imaging is rather time consuming and may delay patient flow.

## Conclusions

NIR is a non-invasive imaging modality commonly performed alongside OCT. In this study, we have shown NIR proved useful to evaluate NVCs in PDR and may obviate the need of additional testing. Specifically, observing the changes in neovascularization contrast and reflectivity due to perfusion of the neovascular tissue, can aid clinicians in daily practice by helping in the detection, assessment of activity and monitoring of proliferative diabetic disease.

## Data Availability

The data is available from the corresponding author upon reasonable request.

## Abbreviations

DM: Diabetes mellitus
DR: Diabetic retinopathy
FA: Fluorescein angiography
ILM: Internal limiting membrane
NIR: Near-infrared reflectance
NVC: Neovascular complex
NVD: Neovascularization of the disc
NVE: Neovascularization elsewhere
OCT: Optical coherence tomography
OCTA: Optical coherence tomography angiography
PDR: Proliferative diabetic retinopathy
VEGF: Vascular endothelial growth factor
